# (2,2′-Bipyridine-κ^2^ *N*,*N*′)chlorido(2-hy­droxy-2,2-diphenyl­acetato-κ^2^ *O* ^1^,*O* ^1′^)copper(II)

**DOI:** 10.1107/S160053681100729X

**Published:** 2011-03-09

**Authors:** Md. Yeamin Reza, Laila Arjuman Banu, M. Saidul Islam, Seik Weng Ng, Edward R. T. Tiekink

**Affiliations:** a Department of Chemistry, Rajshahi University, Bangladesh; b Department of Chemistry, University of Malaya, 50603 Kuala Lumpur, Malaysia

## Abstract

The Cu(II) atom in the title complex, [Cu(C_14_H_11_O_3_)Cl(C_10_H_8_N_2_)], exists within a ClN_2_O_2_ donor set defined by a chloride ion, an asymmetrically chelating carboxyl­ate ligand, and a symmetrically chelating 2,2′-bipyridine mol­ecule. The coordination geometry is square pyramidal with the axial site occupied by the O atom forming the weaker Cu—O inter­action. The hy­droxy group forms an intra­molecular hydrogen bond with the axial O atom, as well as an inter­molecular O—H⋯Cl hydrogen bond. The latter leads to the formation of [100] supra­molecular chains in the crystal, with the Cu(II) atoms lying in a line.

## Related literature

For recent structural studies on metal complexes of anions derived from benzilic acid, see: Yang *et al.* (2010 ▶); Reza *et al.* (2010 ▶). For additional structural analysis, see: Addison *et al.* (1984 ▶); Spek (2009 ▶).
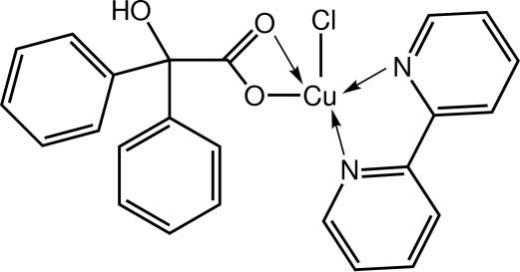


## Experimental

### 

#### Crystal data

[Cu(C_14_H_11_O_3_)Cl(C_10_H_8_N_2_)]
*M* *_r_* = 482.40
Monoclinic, 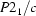

*a* = 7.1537 (9) Å
*b* = 15.7277 (19) Å
*c* = 18.601 (4) Å
β = 97.806 (14)°
*V* = 2073.5 (5) Å^3^
*Z* = 4
Mo *K*α radiation
μ = 1.21 mm^−1^
*T* = 293 K
0.20 × 0.15 × 0.10 mm

#### Data collection

Agilent SuperNova Dual diffractometer with an Atlas detector
Absorption correction: multi-scan (*CrysAlis PRO*; Agilent, 2010 ▶) *T*_min_ = 0.571, *T*_max_ = 1.000
8454 measured reflections
3651 independent reflections
2719 reflections with *I* > 2σ(*I*)
*R*_int_ = 0.053

#### Refinement

*R*[*F* ^2^ > 2σ(*F* ^2^)] = 0.060
*wR*(*F* ^2^) = 0.238
*S* = 1.03
3651 reflections
281 parameters
H-atom parameters constrained
Δρ_max_ = 0.91 e Å^−3^
Δρ_min_ = −1.42 e Å^−3^

### 

Data collection: *CrysAlis PRO* (Agilent, 2010 ▶); cell refinement: *CrysAlis PRO*; data reduction: *CrysAlis PRO*; program(s) used to solve structure: *SHELXS97* (Sheldrick, 2008 ▶); program(s) used to refine structure: *SHELXL97* (Sheldrick, 2008 ▶); molecular graphics: *ORTEP-3* (Farrugia, 1997 ▶) and *DIAMOND* (Brandenburg, 2006 ▶); software used to prepare material for publication: *publCIF* (Westrip, 2010 ▶).

## Supplementary Material

Crystal structure: contains datablocks global, I. DOI: 10.1107/S160053681100729X/hb5805sup1.cif

Structure factors: contains datablocks I. DOI: 10.1107/S160053681100729X/hb5805Isup2.hkl

Additional supplementary materials:  crystallographic information; 3D view; checkCIF report

## Figures and Tables

**Table 1:** Selected bond lengths (Å)

Cu—Cl1	2.2301 (18)
Cu—O1	1.971 (4)
Cu—O2	2.476 (4)
Cu—N1	2.006 (5)
Cu—N2	1.976 (5)

**Table 2:** Hydrogen-bond geometry (Å, °)

*D*—H⋯*A*	*D*—H	H⋯*A*	*D*⋯*A*	*D*—H⋯*A*
O3—H3*o*⋯O2	0.82	2.19	2.622 (6)	113
O3—H3*o*⋯Cl1^i^	0.82	2.62	3.328 (5)	146

Symmetry code: (i) 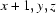.

**Fig. 1.**
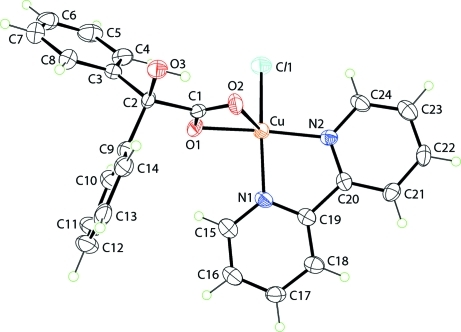
Molecular structure of (I), showing displacement ellipsoids at the 50% probability level.

**Fig. 2.**
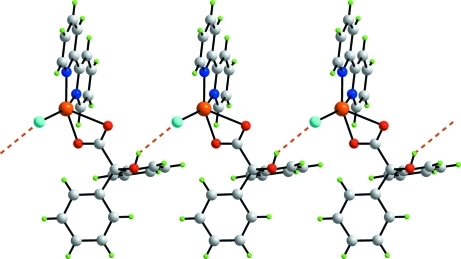
Supramolecular chain along the a axis in (I) mediated by O—H···Cl hydrogen bonds (shown as orange dashed lines).

## supplementary crystallographic information

### Comment

Recent structural investigations of benzilate complexes have confirmed that anions derived from benzilic acid can function as multidentate ligands with versatile coordination modes (Reza *et al.*, 2010; Yang *et al.*, 2010). Herein, the crystal and molecular structure of a mononuclear Cu^II^ complex, (I), is described.

The Cu atom in (I) is coordinated by a Cl, an asymmetrically chelating carboxylate anion, and a symmetrically chelating 2,2'-bipyridine ligand, Table 1. The asymmetric mode of coordination of the carboxylate is reflected in the disparate C—O bond distances with the longer C1—O1 distance [1.285 (8) Å] being associated with the shorter Cu—O1 interaction, and the short C1—O2 distance [1.204 (7) Å] associated with the weaker Cu—O2 contact. The resultant ClN_2_O_2_ donor set defines a square pyramid. This assignment is based on the value calculated for τ of 0.07 for the Cu atom, which compares to the τ values of 0.0 and 1.0 for ideal square pyramidal and trigonal bi-pyramidal geometries, respectively (Spek, 2009; Addison *et al.*, 1984). In this description, the weakly coordinating O2 atom defines the axial site. While not participating in direct coordination to the Cu atom, the hydroxyl group forms an intramolecular hydrogen bond with the O2 atom as well as an intermolecular O—H···Cl hydrogen bond, Table 2. The latter leads to the generation of supramolecular chains along the *a* axis, Fig. 2, whereby the Cu atoms lie on a line.

### Experimental

A mixture of copper chloride (0.134 g,1 mmol), benzilic acid (0.228 g, 1 mmol), 2,2'-bipyridine (0.196 g, 1 mmol) and Et_3_N (0.1 g, 1 mmol) was placed into methanol (40 ml) and the resultant solution was heated to 323 K for 0.5 h. Initial precipitates were filtered off and the filtrate was allowed to stand for several days. Blue blocks of the title compound were collected, washed with methanol and air-dried at room temperature. *M*. pt. 457 K.

### Refinement

The O– and C-bound H atoms were geometrically placed (O—H = 0.82 Å and C–H = 0.93 Å) and refined as riding with *U*_iso_(H) = *zU*_eq_(carrier atom); *z* = 1.5 for O and *z* = 1.2 for C. The maximum and minimum residual electron density peaks of 0.91 and 1.42 e Å^-3^, respectively, were located 0.93 Å and 0.78 Å from the N1 and Cu atoms, respectively.

### Crystal data

**Table:**

[Cu(C_14_H_11_O_3_)Cl(C_10_H_8_N_2_)]	*F*(000) = 988
*M**_r_* = 482.40	*D*_x_ = 1.545 Mg m^−^^3^
Monoclinic, *P*2_1_/*c*	Mo *K*α radiation, λ = 0.71073 Å
Hall symbol: -P 2ybc	Cell parameters from 3252 reflections
*a* = 7.1537 (9) Å	θ = 2.6–29.4°
*b* = 15.7277 (19) Å	µ = 1.21 mm^−^^1^
*c* = 18.601 (4) Å	*T* = 293 K
β = 97.806 (14)°	Block, blue
*V* = 2073.5 (5) Å^3^	0.20 × 0.15 × 0.10 mm
*Z* = 4	

### Data collection

**Table:**

Agilent SuperNova Dual diffractometer with an Atlas detector	3651 independent reflections
Radiation source: SuperNova (Mo) X-ray Source	2719 reflections with *I* > 2σ(*I*)
Mirror	*R*_int_ = 0.053
Detector resolution: 10.4041 pixels mm^-1^	θ_max_ = 25.0°, θ_min_ = 2.6°
ω scans	*h* = −8→8
Absorption correction: multi-scan (*CrysAlis PRO*; Agilent, 2010)	*k* = −18→17
*T*_min_ = 0.571, *T*_max_ = 1.000	*l* = −21→22
8454 measured reflections	

### Refinement

**Table:**

Refinement on *F*^2^	Primary atom site location: structure-invariant direct methods
Least-squares matrix: full	Secondary atom site location: difference Fourier map
*R*[*F*^2^ > 2σ(*F*^2^)] = 0.060	Hydrogen site location: inferred from neighbouring sites
*wR*(*F*^2^) = 0.238	H-atom parameters constrained
*S* = 1.03	*w* = 1/[σ^2^(*F*_o_^2^) + (0.1324*P*)^2^ + 7.2136*P*] where *P* = (*F*_o_^2^ + 2*F*_c_^2^)/3
3651 reflections	(Δ/σ)_max_ < 0.001
281 parameters	Δρ_max_ = 0.91 e Å^−^^3^
0 restraints	Δρ_min_ = −1.42 e Å^−^^3^

### Special details

**Table:**

Geometry. All s.u.'s (except the s.u. in the dihedral angle between two l.s. planes) are estimated using the full covariance matrix. The cell s.u.'s are taken into account individually in the estimation of s.u.'s in distances, angles and torsion angles; correlations between s.u.'s in cell parameters are only used when they are defined by crystal symmetry. An approximate (isotropic) treatment of cell s.u.'s is used for estimating s.u.'s involving l.s. planes.
Refinement. Refinement of *F*^2^ against ALL reflections. The weighted *R*-factor *wR* and goodness of fit *S* are based on *F*^2^, conventional *R*-factors *R* are based on *F*, with *F* set to zero for negative *F*^2^. The threshold expression of *F*^2^ > 2σ(*F*^2^) is used only for calculating *R*-factors(gt) *etc*. and is not relevant to the choice of reflections for refinement. *R*-factors based on *F*^2^ are statistically about twice as large as those based on *F*, and *R*- factors based on ALL data will be even larger.

### Fractional atomic coordinates and isotropic or equivalent isotropic displacement parameters (Å^2^)

**Table:**

	*x*	*y*	*z*	*U*_iso_*/*U*_eq_	
Cu	0.63293 (10)	0.44986 (4)	0.36367 (4)	0.0351 (3)	
Cl1	0.4262 (2)	0.34756 (11)	0.32669 (10)	0.0475 (5)	
N1	0.7215 (8)	0.5629 (3)	0.4053 (3)	0.0390 (13)	
N2	0.6723 (7)	0.4182 (3)	0.4674 (3)	0.0337 (11)	
O1	0.6859 (6)	0.4804 (3)	0.2657 (2)	0.0401 (10)	
O2	0.9191 (6)	0.3986 (3)	0.3158 (2)	0.0415 (11)	
O3	1.0661 (7)	0.3757 (3)	0.1956 (2)	0.0438 (11)	
H3o	1.1135	0.3685	0.2378	0.066*	
C1	0.8391 (9)	0.4378 (4)	0.2651 (3)	0.0330 (14)	
C2	0.9273 (9)	0.4407 (4)	0.1925 (3)	0.0307 (13)	
C3	0.7914 (9)	0.4235 (4)	0.1234 (3)	0.0350 (13)	
C4	0.6016 (10)	0.4065 (4)	0.1209 (4)	0.0457 (16)	
H4	0.5468	0.4054	0.1634	0.055*	
C5	0.4940 (11)	0.3911 (5)	0.0554 (5)	0.058 (2)	
H5	0.3653	0.3811	0.0536	0.070*	
C6	0.5752 (13)	0.3905 (5)	−0.0080 (4)	0.062 (2)	
H6	0.5008	0.3794	−0.0520	0.074*	
C7	0.7616 (13)	0.4058 (5)	−0.0067 (4)	0.061 (2)	
H7	0.8161	0.4044	−0.0493	0.073*	
C8	0.8696 (11)	0.4236 (5)	0.0586 (4)	0.0490 (17)	
H8	0.9971	0.4358	0.0596	0.059*	
C9	1.0175 (8)	0.5291 (4)	0.1890 (3)	0.0342 (13)	
C10	0.9071 (10)	0.6006 (4)	0.1767 (4)	0.0414 (15)	
H10	0.7765	0.5952	0.1684	0.050*	
C11	0.9891 (12)	0.6816 (4)	0.1764 (4)	0.0550 (19)	
H11	0.9129	0.7295	0.1691	0.066*	
C12	1.1818 (12)	0.6901 (5)	0.1871 (4)	0.057 (2)	
H12	1.2369	0.7435	0.1861	0.068*	
C13	1.2907 (10)	0.6200 (5)	0.1991 (3)	0.0491 (18)	
H13	1.4213	0.6259	0.2060	0.059*	
C14	1.2124 (9)	0.5387 (4)	0.2013 (4)	0.0413 (15)	
H14	1.2900	0.4915	0.2108	0.050*	
C15	0.7393 (9)	0.6339 (4)	0.3682 (4)	0.0448 (16)	
H15	0.7052	0.6331	0.3182	0.054*	
C16	0.8060 (10)	0.7086 (4)	0.4006 (4)	0.0466 (16)	
H16	0.8175	0.7571	0.3730	0.056*	
C17	0.8541 (10)	0.7101 (4)	0.4732 (4)	0.0464 (16)	
H17	0.8988	0.7599	0.4964	0.056*	
C18	0.8367 (9)	0.6366 (4)	0.5133 (4)	0.0410 (15)	
H18	0.8700	0.6364	0.5634	0.049*	
C19	0.7685 (8)	0.5635 (4)	0.4772 (4)	0.0339 (14)	
C20	0.7432 (8)	0.4810 (4)	0.5129 (3)	0.0319 (13)	
C21	0.7927 (9)	0.4669 (4)	0.5869 (3)	0.0393 (15)	
H21	0.8404	0.5107	0.6177	0.047*	
C22	0.7690 (10)	0.3860 (4)	0.6135 (3)	0.0431 (16)	
H22	0.8011	0.3748	0.6627	0.052*	
C23	0.6981 (10)	0.3219 (4)	0.5670 (4)	0.0484 (17)	
H23	0.6828	0.2672	0.5844	0.058*	
C24	0.6505 (10)	0.3403 (4)	0.4949 (4)	0.0432 (16)	
H24	0.6012	0.2972	0.4637	0.052*	

### Atomic displacement parameters (Å^2^)

**Table:**

	*U*^11^	*U*^22^	*U*^33^	*U*^12^	*U*^13^	*U*^23^
Cu	0.0404 (5)	0.0315 (5)	0.0357 (5)	0.0002 (3)	0.0137 (4)	−0.0010 (3)
Cl1	0.0425 (9)	0.0453 (9)	0.0547 (11)	−0.0055 (7)	0.0066 (8)	−0.0046 (8)
N1	0.050 (3)	0.027 (2)	0.045 (3)	0.001 (2)	0.025 (3)	−0.001 (2)
N2	0.034 (3)	0.032 (3)	0.037 (3)	−0.003 (2)	0.009 (2)	−0.003 (2)
O1	0.053 (3)	0.042 (2)	0.029 (2)	0.005 (2)	0.018 (2)	−0.0053 (19)
O2	0.050 (3)	0.043 (2)	0.032 (2)	0.003 (2)	0.009 (2)	0.012 (2)
O3	0.058 (3)	0.033 (2)	0.042 (3)	0.017 (2)	0.012 (2)	−0.002 (2)
C1	0.045 (3)	0.026 (3)	0.029 (3)	−0.007 (3)	0.011 (3)	−0.006 (2)
C2	0.040 (3)	0.032 (3)	0.019 (3)	0.005 (2)	0.002 (2)	−0.001 (2)
C3	0.045 (3)	0.025 (3)	0.035 (3)	0.003 (3)	0.005 (3)	−0.001 (3)
C4	0.046 (4)	0.041 (4)	0.052 (4)	−0.005 (3)	0.010 (3)	−0.010 (3)
C5	0.050 (4)	0.045 (4)	0.075 (6)	−0.004 (3)	−0.009 (4)	−0.010 (4)
C6	0.086 (6)	0.043 (4)	0.046 (5)	−0.002 (4)	−0.025 (4)	−0.004 (3)
C7	0.090 (6)	0.053 (4)	0.041 (4)	−0.008 (4)	0.014 (4)	0.001 (3)
C8	0.063 (4)	0.048 (4)	0.037 (4)	−0.005 (4)	0.012 (3)	−0.003 (3)
C9	0.038 (3)	0.033 (3)	0.034 (3)	−0.001 (3)	0.014 (3)	−0.006 (3)
C10	0.043 (3)	0.038 (3)	0.045 (4)	0.003 (3)	0.011 (3)	0.004 (3)
C11	0.070 (5)	0.030 (3)	0.067 (5)	0.002 (3)	0.019 (4)	0.001 (3)
C12	0.075 (5)	0.038 (4)	0.063 (5)	−0.020 (4)	0.030 (4)	−0.007 (3)
C13	0.052 (4)	0.069 (5)	0.028 (3)	−0.021 (4)	0.012 (3)	−0.007 (3)
C14	0.044 (4)	0.044 (4)	0.038 (4)	0.002 (3)	0.013 (3)	−0.002 (3)
C15	0.040 (4)	0.041 (4)	0.053 (4)	0.000 (3)	0.005 (3)	0.009 (3)
C16	0.050 (4)	0.034 (3)	0.058 (5)	−0.001 (3)	0.016 (3)	0.007 (3)
C17	0.058 (4)	0.033 (3)	0.049 (4)	−0.006 (3)	0.011 (3)	−0.008 (3)
C18	0.040 (3)	0.034 (3)	0.051 (4)	0.000 (3)	0.012 (3)	−0.002 (3)
C19	0.028 (3)	0.033 (3)	0.044 (4)	0.002 (2)	0.016 (3)	0.001 (3)
C20	0.028 (3)	0.035 (3)	0.034 (3)	0.005 (2)	0.010 (2)	0.005 (3)
C21	0.043 (4)	0.044 (3)	0.033 (3)	0.004 (3)	0.011 (3)	−0.006 (3)
C22	0.052 (4)	0.052 (4)	0.026 (3)	0.006 (3)	0.008 (3)	0.007 (3)
C23	0.051 (4)	0.040 (4)	0.058 (5)	0.003 (3)	0.021 (3)	0.011 (3)
C24	0.050 (4)	0.030 (3)	0.053 (4)	−0.004 (3)	0.022 (3)	0.004 (3)

### Geometric parameters (Å, °)

**Table:**

Cu—Cl1	2.2301 (18)	C9—C14	1.390 (9)
Cu—O1	1.971 (4)	C10—C11	1.403 (9)
Cu—O2	2.476 (4)	C10—H10	0.9300
Cu—N1	2.006 (5)	C11—C12	1.372 (11)
Cu—N2	1.976 (5)	C11—H11	0.9300
N1—C15	1.329 (8)	C12—C13	1.351 (11)
N1—C19	1.333 (9)	C12—H12	0.9300
N2—C24	1.345 (8)	C13—C14	1.399 (10)
N2—C20	1.353 (8)	C13—H13	0.9300
O1—C1	1.285 (8)	C14—H14	0.9300
O2—C1	1.204 (7)	C15—C16	1.376 (10)
O3—C2	1.421 (7)	C15—H15	0.9300
O3—H3o	0.8200	C16—C17	1.348 (10)
C1—C2	1.567 (8)	C16—H16	0.9300
C2—C3	1.527 (8)	C17—C18	1.390 (9)
C2—C9	1.537 (8)	C17—H17	0.9300
C3—C4	1.379 (9)	C18—C19	1.387 (9)
C3—C8	1.395 (9)	C18—H18	0.9300
C4—C5	1.371 (10)	C19—C20	1.481 (8)
C4—H4	0.9300	C20—C21	1.391 (9)
C5—C6	1.385 (12)	C21—C22	1.384 (9)
C5—H5	0.9300	C21—H21	0.9300
C6—C7	1.352 (12)	C22—C23	1.379 (10)
C6—H6	0.9300	C22—H22	0.9300
C7—C8	1.377 (11)	C23—C24	1.370 (10)
C7—H7	0.9300	C23—H23	0.9300
C8—H8	0.9300	C24—H24	0.9300
C9—C10	1.375 (9)		
			
O1—Cu—N2	160.9 (2)	C10—C9—C2	120.8 (5)
O1—Cu—N1	92.9 (2)	C14—C9—C2	120.6 (5)
N2—Cu—N1	81.4 (2)	C9—C10—C11	120.8 (6)
O1—Cu—Cl1	95.35 (14)	C9—C10—H10	119.6
N2—Cu—Cl1	96.91 (15)	C11—C10—H10	119.6
N1—Cu—Cl1	156.84 (16)	C12—C11—C10	120.0 (7)
O1—Cu—O2	58.31 (16)	C12—C11—H11	120.0
N2—Cu—O2	104.72 (18)	C10—C11—H11	120.0
N1—Cu—O2	101.21 (18)	C13—C12—C11	119.3 (6)
Cl1—Cu—O2	101.55 (12)	C13—C12—H12	120.3
C15—N1—C19	119.0 (6)	C11—C12—H12	120.3
C15—N1—Cu	126.3 (5)	C12—C13—C14	121.7 (7)
C19—N1—Cu	114.6 (4)	C12—C13—H13	119.1
C24—N2—C20	118.7 (6)	C14—C13—H13	119.1
C24—N2—Cu	126.3 (4)	C9—C14—C13	119.5 (6)
C20—N2—Cu	114.8 (4)	C9—C14—H14	120.3
C1—O1—Cu	98.7 (4)	C13—C14—H14	120.3
C1—O2—Cu	77.7 (4)	N1—C15—C16	122.9 (7)
C2—O3—H3o	109.5	N1—C15—H15	118.6
O2—C1—O1	125.2 (6)	C16—C15—H15	118.6
O2—C1—C2	119.0 (5)	C17—C16—C15	118.7 (6)
O1—C1—C2	115.8 (5)	C17—C16—H16	120.7
O3—C2—C3	105.5 (4)	C15—C16—H16	120.7
O3—C2—C9	111.0 (5)	C16—C17—C18	119.6 (6)
C3—C2—C9	110.4 (5)	C16—C17—H17	120.2
O3—C2—C1	107.8 (5)	C18—C17—H17	120.2
C3—C2—C1	115.8 (5)	C19—C18—C17	118.7 (6)
C9—C2—C1	106.4 (4)	C19—C18—H18	120.7
C4—C3—C8	118.6 (6)	C17—C18—H18	120.7
C4—C3—C2	125.1 (6)	N1—C19—C18	121.1 (6)
C8—C3—C2	116.3 (6)	N1—C19—C20	114.5 (5)
C3—C4—C5	119.8 (7)	C18—C19—C20	124.4 (6)
C3—C4—H4	120.1	N2—C20—C21	121.8 (6)
C5—C4—H4	120.1	N2—C20—C19	114.6 (5)
C4—C5—C6	120.5 (7)	C21—C20—C19	123.6 (6)
C4—C5—H5	119.7	C22—C21—C20	118.2 (6)
C6—C5—H5	119.7	C22—C21—H21	120.9
C7—C6—C5	120.7 (7)	C20—C21—H21	120.9
C7—C6—H6	119.7	C23—C22—C21	120.1 (6)
C5—C6—H6	119.7	C23—C22—H22	119.9
C6—C7—C8	119.1 (8)	C21—C22—H22	119.9
C6—C7—H7	120.5	C24—C23—C22	118.7 (6)
C8—C7—H7	120.5	C24—C23—H23	120.6
C7—C8—C3	121.3 (7)	C22—C23—H23	120.6
C7—C8—H8	119.4	N2—C24—C23	122.6 (6)
C3—C8—H8	119.4	N2—C24—H24	118.7
C10—C9—C14	118.6 (6)	C23—C24—H24	118.7
			
O1—Cu—N1—C15	19.3 (6)	C5—C6—C7—C8	−1.1 (12)
N2—Cu—N1—C15	−179.0 (6)	C6—C7—C8—C3	1.9 (12)
Cl1—Cu—N1—C15	−91.6 (6)	C4—C3—C8—C7	−1.0 (10)
O2—Cu—N1—C15	77.6 (5)	C2—C3—C8—C7	177.7 (6)
O1—Cu—N1—C19	−159.5 (4)	O3—C2—C9—C10	171.8 (5)
N2—Cu—N1—C19	2.2 (4)	C3—C2—C9—C10	55.3 (7)
Cl1—Cu—N1—C19	89.6 (6)	C1—C2—C9—C10	−71.2 (7)
O2—Cu—N1—C19	−101.2 (4)	O3—C2—C9—C14	−10.8 (8)
O1—Cu—N2—C24	−103.3 (7)	C3—C2—C9—C14	−127.3 (6)
N1—Cu—N2—C24	−177.1 (5)	C1—C2—C9—C14	106.2 (6)
Cl1—Cu—N2—C24	26.2 (5)	C14—C9—C10—C11	−0.1 (10)
O2—Cu—N2—C24	−77.7 (5)	C2—C9—C10—C11	177.4 (6)
O1—Cu—N2—C20	70.8 (7)	C9—C10—C11—C12	1.4 (11)
N1—Cu—N2—C20	−3.0 (4)	C10—C11—C12—C13	−1.2 (12)
Cl1—Cu—N2—C20	−159.7 (4)	C11—C12—C13—C14	−0.3 (11)
O2—Cu—N2—C20	96.4 (4)	C10—C9—C14—C13	−1.4 (9)
N2—Cu—O1—C1	31.7 (7)	C2—C9—C14—C13	−178.9 (6)
N1—Cu—O1—C1	103.6 (4)	C12—C13—C14—C9	1.6 (10)
Cl1—Cu—O1—C1	−98.1 (3)	C19—N1—C15—C16	0.6 (10)
O2—Cu—O1—C1	2.2 (3)	Cu—N1—C15—C16	−178.2 (5)
O1—Cu—O2—C1	−2.4 (3)	N1—C15—C16—C17	−0.5 (10)
N2—Cu—O2—C1	−172.8 (4)	C15—C16—C17—C18	0.4 (10)
N1—Cu—O2—C1	−88.9 (4)	C16—C17—C18—C19	−0.4 (10)
Cl1—Cu—O2—C1	86.8 (3)	C15—N1—C19—C18	−0.6 (9)
Cu—O2—C1—O1	3.8 (5)	Cu—N1—C19—C18	178.4 (4)
Cu—O2—C1—C2	−177.9 (5)	C15—N1—C19—C20	−179.9 (5)
Cu—O1—C1—O2	−4.7 (7)	Cu—N1—C19—C20	−1.0 (6)
Cu—O1—C1—C2	177.0 (4)	C17—C18—C19—N1	0.5 (9)
O2—C1—C2—O3	15.2 (7)	C17—C18—C19—C20	179.8 (6)
O1—C1—C2—O3	−166.4 (5)	C24—N2—C20—C21	−0.3 (8)
O2—C1—C2—C3	133.0 (6)	Cu—N2—C20—C21	−174.9 (4)
O1—C1—C2—C3	−48.6 (7)	C24—N2—C20—C19	177.8 (5)
O2—C1—C2—C9	−103.9 (6)	Cu—N2—C20—C19	3.3 (6)
O1—C1—C2—C9	74.5 (6)	N1—C19—C20—N2	−1.4 (7)
O3—C2—C3—C4	119.4 (6)	C18—C19—C20—N2	179.2 (5)
C9—C2—C3—C4	−120.7 (6)	N1—C19—C20—C21	176.7 (6)
C1—C2—C3—C4	0.3 (8)	C18—C19—C20—C21	−2.7 (9)
O3—C2—C3—C8	−59.2 (7)	N2—C20—C21—C22	0.5 (9)
C9—C2—C3—C8	60.8 (7)	C19—C20—C21—C22	−177.5 (6)
C1—C2—C3—C8	−178.3 (5)	C20—C21—C22—C23	0.0 (10)
C8—C3—C4—C5	−0.8 (10)	C21—C22—C23—C24	−0.6 (10)
C2—C3—C4—C5	−179.3 (6)	C20—N2—C24—C23	−0.3 (9)
C3—C4—C5—C6	1.6 (11)	Cu—N2—C24—C23	173.6 (5)
C4—C5—C6—C7	−0.7 (12)	C22—C23—C24—N2	0.8 (10)

### Hydrogen-bond geometry (Å, °)

**Table:**

*D*—H···*A*	*D*—H	H···*A*	*D*···*A*	*D*—H···*A*
O3—H3o···O2	0.82	2.19	2.622 (6)	113
O3—H3o···Cl1^i^	0.82	2.62	3.328 (5)	146

Symmetry codes: (i) *x*+1, *y*, *z*.
